# Comparative interference by haemolysis in automated assays for bilirubin at multiple analyte concentrations

**DOI:** 10.1155/S1463924689000490

**Published:** 1989

**Authors:** Robert A. Webster, Peter Molnar, Stephen E. Kahn

**Affiliations:** 1 Department of Pathology Loyala University Medical Center Maywood Illinois 60153 USA; 2 Department of Biochemistry and Office of Consolidated Laboratory Services—Clinical Chemistry Rush- Presbyterian-St Luke's Medical Center Chicago Illinois 60612 USA

## Abstract

The negative interference caused by haemolysis in manual bilirubin assays contrasts with the positive interference reported for some automated methods utilizing the same basic chemistry. A comparison was therefore made of the haemolysis interference associated with several automated bilirubin methodologies: multilayer film (Kodak Ektachem- total bilirubin (TBil), direct bilirubin (DBil), conjugated bilirubin (Bc), unconjugated bilirubin (Bu),; tableted reagents (Baxter Paramax- TBil, DBil); continuous flow (Technicon SMAC — TBil). Thirty serum pools were analysed (five concentrations of bilirubin, 2-229 μmol/l; six concentrations of haemoglobin, 0.00002-0.052 mmol/1). All methods, except one (Bc), exhibited both positive and negative interference, depending upon the relative haemoglobin and bilirubin concentrations. This interference, at any given haemoglobin concentration, was neither constant nor proportional with increasing bilirubin concentration. These complex patterns of interference suggest that the best characterization of interference is obtained when (1) both percentage-difference and absolute-difference ‘interferographs’ are plotted; and (2) the interference is determined at multiple analyte concentrations.


Journal of Automatic Chemistry Vol. 11, No. 6 (November–December 1989), p. 254–259

### Comparative interference by haemolysis in automated assays for bilirubin at multiple analyte concentrations

**Robert A. Webster†, Peter Molnar and Stephen E. Kahn‡**
*Department of Pathology, Loyala University Medical Center, Maywood, Illinois 60153, USA*

*The negative interference caused by haemolysis in manual bilirubin assays contrasts with the positive interference reported for some automated methods utilizing the same basic chemistry. A comparison was therefore made of the haemolysis interference associated with several automated bilirubin methodologies: multi-layer film (Kodak Ektachem – total bilirubin (TBil), direct bilirubin (DBil), conjugated bilirubin (Bc), unconjugated bilirubin (Bu),; tableted reagents (Baxter Paramax – TBil, DBil); continuous flow (Technicon SMAC – TBil). Thirty serum pools were analysed (five concentrations of bilirubin, 2–229 µmol/l; six concentrations of haemoglobin, 0·00002–0·052 mmol/l). All methods, except one (Bc), exhibited both positive and negative interference, depending upon the relative haemoglobin and bilirubin concentrations. This interference, at any given haemoglobin concentration, was neither constant nor proportional with increasing bilirubin concentration. These complex patterns of interference suggest that the best characterization of interference is obtained when (1) both percentage-difference and absolute-difference 'interferographs' are plotted; and (2) the interference is determined at multiple analyte concentrations.*

### Introduction

Haemolysis causes negative interference in manual methods for the assay of bilirubin that are based upon the formation of an azobilirubin chromophore [1, 2]. Unless stated otherwise in the technical manuals for automated instruments, one might be tempted to assume that haemolysis causes negative interference in automated bilirubin methods that have the same chemical basis as the manual methods. This is not necessarily the case, however, with positive interference being reported for some such automated bilirubin assays [3]. In addition, this positive interference can be quite substantial. This prompted the authors to compare the interference caused by varying degrees of haemolysis in several automated methods at multiple bilirubin concentrations. In addition, two formats for the graphical presentation of interference data have been compared: as a standard 'interferograph' (plot of percentage difference of the test result from the control result versus increasing interferant concentration) [3], and as a modified interferograph (plot of absolute difference of the test result from the control result versus increasing interferant concentration).

### Materials and methods

Bilirubin was assayed by three different automated methodologies: multilayer film (Ektachem 700, Eastman Kodak Co., Rochester, New York, USA), tableted reagents (Paramax, Baxter Healthcare Corp., Paramax Systems Division, Santa Ana, California, USA) and continuous flow (SMAC, Technicon Instruments Corp., Tarrytown, New York, USA). Total bilirubin (TBil), conjugated bilirubin (Bc), and unconjugated bilirubin (Bu) were measured with the Ektachem, with direct bilirubin (DBil) being calculated (TBil minus Bu). TBil and DBil were measured with the Paramax, while only TBil was measured with the SMAC.

Four replicate bilirubin determinations were carried out on 30 serum pools, which included five concentrations of bilirubin (TBil 2–229 µmol/l) and six concentrations of haemoglobin (0·00002–0·052 mmol/l), representing six levels of haemolysis (0, trace, 1+, 2+, 3+, 4+) [4]. The replicate assays were done on different days with all three instruments.

The 30 serum pools were prepared from two stock pools, with TBil concentrations of 3 and 650 µmol/l (as determined on Paramax), and a stock haemolysate. The two stock serum pools were prepared by combining the unused portions of specimens that had been received by the laboratory for analysis. These stock pools were stored frozen (−20 °C) until a sufficient total volume was obtained for the preparation of the 30 pools for analysis. At this point, the stock pools were thawed, filtered (Whatman No. 1 paper), and assayed for TBil. The two stock pools and the stock haemolysate (see below) were combined in varying proportions to obtain the 30 pools for analysis. Each of these was divided into 1·3 ml aliquots that were stored frozen (−20 °C) until analysed. Each aliquot was thawed only once prior to analysis for bilirubin or haemoglobin (see below). Throughout the storage, preparation and analysis of the serum pools, every effort was made to protect them from light.

The stock haemolysate was prepared from heparinized blood from one normal individual. The erythrocytes were separated from plasma by centrifugation at room temperature and then washed three times with isotonic saline. Lysis was accomplished by suspending the final pellet of washed erythrocytes in an equal volume of distilled

† Present address: Department of Biochemistry and Office of Consolidated Laboratory Services—Clinical Chemistry, Rush-Presbyterian-St Luke's Medical Center, Chicago, Illinois 60612, USA.

‡ To whom correspondence should be addressed.

0142–0453/89 $3.00 © 1989 Taylor & Francis Ltd.

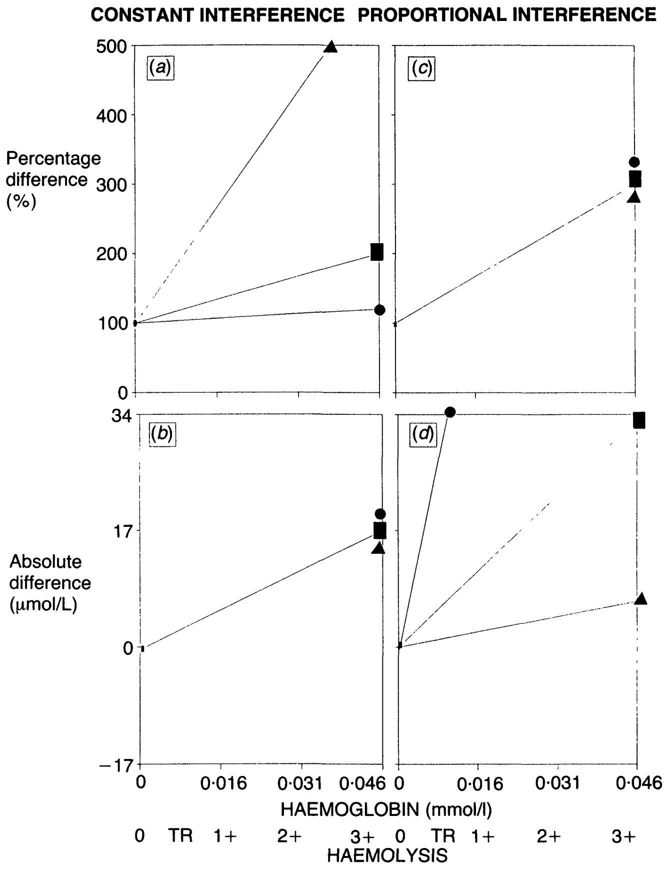

*Figure 1. Hypothetical positive interference of increasing amounts of haemolysate on bilirubin results at multiple bilirubin concentrations. (a) and (b) represent only constant interference, while (c) and (d) represent only proportional interference. The hypothetical control (i.e., without added haemolysate) bilirubin concentrations (μmol/l) were as follows: 3 (▲), 17 (■), 86 (●). TR = trace haemolysis. (a) and (c): percentage difference = 100 × (result with added haemolysate (i.e., test result) divided by result without added haemolysate (i.e., control result)). The control result itself, therefore, is seen as a difference of 100%. (b) and (d): absolute difference = (result with added haemolysate (i.e., test result) minus result wihout added haemolysate (i.e., control result)). The control result itself, therefore, is seen as a difference of 0 μmol/l.*

water, freezing (−20° C) the suspension overnight, and then thawing it at room temperature. The lysate was centrifuged for 30 min at room temperature to pellet the stroma. The supernatant liquid, the haemolysate, was decanted and an aliquot assayed for haemoglobin upon dilution. The haemolysate was divided into 1·0 ml aliquots that were stored frozen (−20 °C) until used.

Total haemoglobin was assayed by a modification of the method of Drabkin [5], in which 0·2 ml of sample was mixed with 2·5 ml of reagent. The standard curve was obtained using Hemoglobinometer Calibrator/Controls (Coulter Diagnostics, Hialeah, Florida, USA) corresponding to haemoglobin concentrations of 0·019, 0·034, and 0·065 mmol/l. The absorbance at 540 nm was determined with a Gilford Response Spectrophotometer (Gilford Instrument Laboratories Inc., Oberlin, Ohio, USA).

Since the amount of oxygenated haemoglobin in a haemolysate decreases during storage, it was important to ensure that the majority of the total haemoglobin was oxygenated and that all the pools had approximately the same content of oxygenated haemoglobin. The latter was determined by a spectrophotometric scanning method [6,7] using the same spectrophotometer as above. Total and oxygenated haemoglobin concentrations were determined in triplicate for each of the 30 serum pools. Oxygenated haemoglobin represented 75·7 ± 2·8% of the total haemoglobin, which was considered acceptable.

The interference data (means) were plotted in two graphical formats. One was a standard, percentage-difference interferograph [3], and the other was an absolute-difference interferograph. For illustrative purposes, these two formats are compared in figure 1 for hypothetical positive interference that is either only constant or only proportional. With constant interference, the absolute amount is dependent only on the interferant concentration and, thus, is independent of the analyte concentration. The percentage difference, therefore, varies with different analyte concentrations. With proportional interference, the absolute amount is dependent on both the interferant and analyte concentrations, such that the percentage difference does not vary with different analyte concentrations.

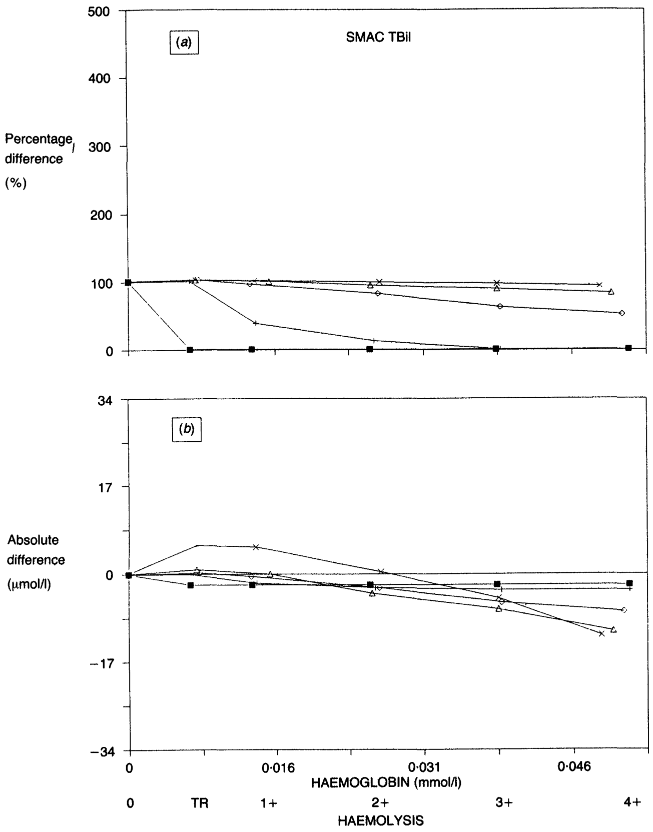

*Figure 2. Effect of increasing amounts of haemolysate on SMAC TBil results at multiple TBil concentrations. The control (i.e., without added haemolysate) TBil concentrations (μmol/l) were as follows: 2 (■), 3 (+), 15 (♦), 63 (▲), 205 (×). Other details are described in the legend of figure 1.*

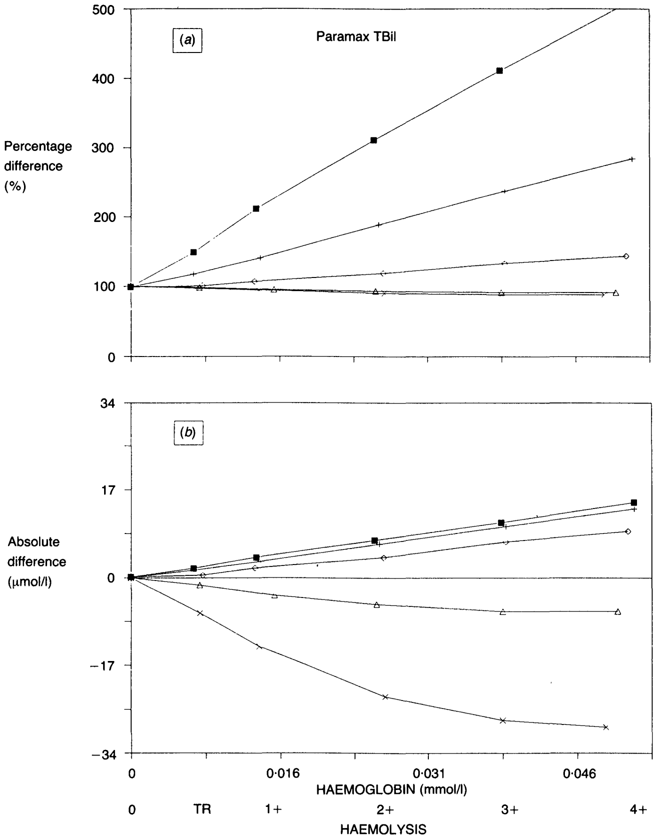

*Figure 3. Effect of increasing amounts of haemolysate on Paramax TBil results at multiple TBil concentrations. The control (i.e., without added haemolysate) TBil concentrations (µmol/l) were as follows: 3(■), 7 (+), 21 (♦), 72 (▲), 229 (×). Other details are described in the legend of figure 1.*

### Results

Figure 2 shows that added haemolysate caused negative interference in the SMAC TBil assay, except at the highest bilirubin concentration and the lowest haemoglobin concentrations, in which cases the interference was positive (figure 2[*b*]). These results also demonstrate that, at any given haemoglobin concentration, the interference was neither constant nor proportional with increasing bilirubin concentration.

With the Paramax TBil method, as shown in figure 3, the haemolysate interference was positive at low bilirubin concentrations and negative at high bilirubin concentrations. This is most apparent in figure 3(*b*), where the absolute difference is plotted versus increasing haemoglobin concentration. The bilirubin concentration at which no interference would be observed ranged between 29 and 43 µmol/l, depending on the haemoglobin concentration. This was determined by plotting the absolute difference versus the logarithm of the control result (not shown). Again, at any given haemoglobin concentration, the interference was neither constant nor proportional with increasing bilirubin concentration.

Similar results were obtained with the Ektachem TBil assay (figure 4), except that the ranges of differences (percentage and absolute) were less than with the Paramax TBil assay. This was also true for the Paramax DBil assay (figure 5), which exhibited a similar pattern of interference. The range of bilirubin concentrations for which no interference would be expected, again depending on the haemoglobin concentration, was 80 to 133 µmol/l for the Ektachem TBil assay and 26 to 53 µmol/l for the Paramax DBil assay (determined as described above for the Paramax TBil assay).

Added haemolysate caused positive interference with the Ektachem DBil results (figure 6), except for negative interference at the highest bilirubin concentration and the lowest haemoglobin concentrations (figure 6[*b*]). This pattern is essentially opposite to that obtained with the SMAC TBil results (figure 2[*b*]). When the interference with the Ektachem DBil results is plotted as the percentage difference (figure 6[*a*]), the range of differences is greater than that for the Paramax TBil, Ektachem Tbil and Paramax DBil results (figures 3[*a*], 4[*a*] and 5[*a*], respectively). On the other hand, when the interference with the Ektachem DBil results is plotted as the absolute difference (figure 6[*b*]), the range of differences is less than that for the Paramax TBil, Ektachem TBil and Paramax DBil results (figures 3[*b*], 4[*b*] and 5[*b*], respectively). Although this discrepancy is due primarily to the lower absolute values of the low Ektachem Dbil control results (i.e., 2 and 3 µmol/l), it suggests that the most accurate picture of interference is obtained when the

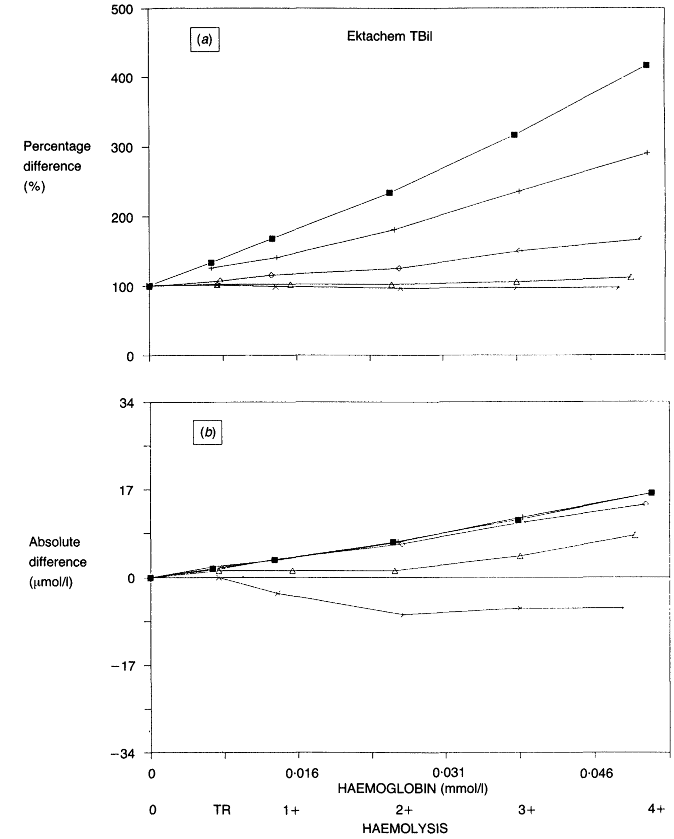

*Figure 4. Effect of increasing amounts of haemolysate on Ektachem TBil results at multiple TBil concentrations. The control (i.e., without added haemolysate) TBil concentrations (µmol/l) were as follows: 5 (■), 9 (+), 21 (♦), 68 (▲), 218 (×). Other details are described in the legend of figure 1.*

absolute difference, rather than the percentage difference, is plotted versus increasing interferant concentration. As with all the previous assays, the interference was neither constant nor proportional.

Figure 7 shows that haemolysate interference in the Ektachem Bu method was primarily negative, except for positive interference at low bilirubin and low haemoglobin concentrations. Although the overall interference appears quite minimal, the highest bilirubin concentration attained was considerably less than those attained with the other methods (62 versus 147–229 μmol/l).

While the interference with the Ektachem Bu method at any given haemoglobin concentration was neither constant nor proportional with increasing bilirubin concentration (see figure 7), the interference with the Ektachem Bc method appeared to be almost constant (figure 8). Again, however, the highest bilirubin concentration attained (101 μmol/l) was somewhat less than those attained with the other methods, except the Ektachem Bu method. With the Ektachem Bc method, only positive haemolysate interference was observed.

The general patterns of interference for all of the methods are summarized and compared in the table.

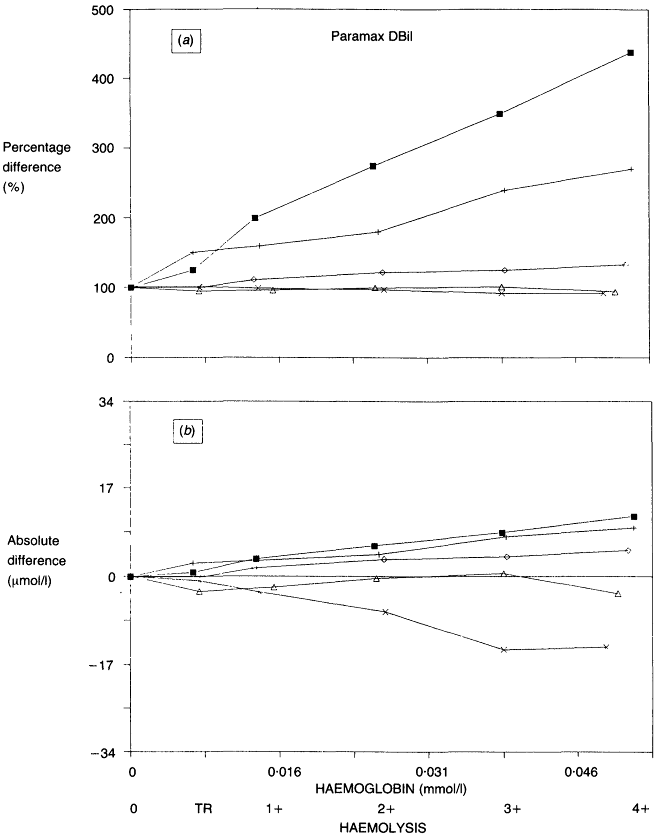

*Figure 5. Effect of increasing amounts of haemolysate on Paramax DBil results at multiple DBil concentrations. The control (i.e., without added haemolysate) DBil concentrations (μmol/l) were as follows: 3 (■), 7 (+), 15 (♦), 51 (▲), 147 (×). Other details are described in the legend of figure 1.*

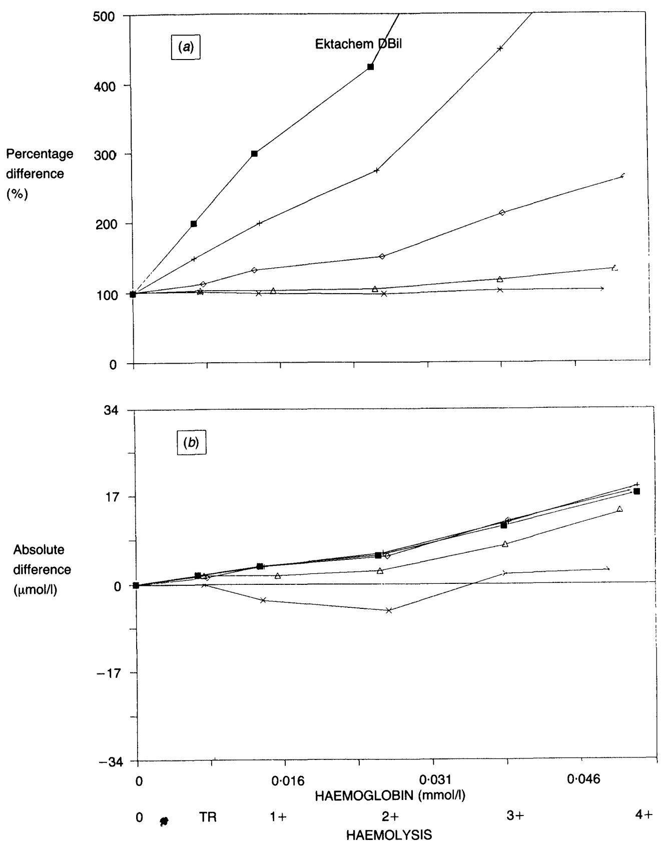

*Figure 6. Effect of increasing amounts of haemolysate on Paramax DBil results at multiple DBil concentrations. The control (i.e., without added haemolysate) DBil concentration (μmol/l) were as follows: 2 (■), 3 (+), 10 (♦), 44 (▲), 154 (×). Other details are described in the legend of figure 1.*

### Discussion

All of the automated bilirubin methods examined, except one, were subject to haemolysis interference that was both positive and negative, depending upon the relative haemoglobin and bilirubin concentrations. The one exception was the Ektachem Bc method, which exhibited only positive interference. For all of the methods, at any given haemoglobin concentration, the interference was neither constant nor proportional with increasing bilirubin concentration. Interference that appeared almost constant was demonstrated with the Ektachem Bc method.

Chemical and/or spectral explanations for these complex patterns of interference were not pursued in this study. Shull *et al.* studied the negative haemolysate interference in manual methods for the assay of total bilirubin. They concluded for the Malloy-Evelyn method that the interference was due to oxidative destruction of the bilirubin [1]. For the Jendrassik-Grof method, the problem appeared to be oxidative destruction of the azobilirubin [2].

Manufacturers' descriptions of the interference due to haemolysis were varied for the methods examined here. No information on haemolysis interference was provided for the SMAC TBil method. For the Paramax TBil and

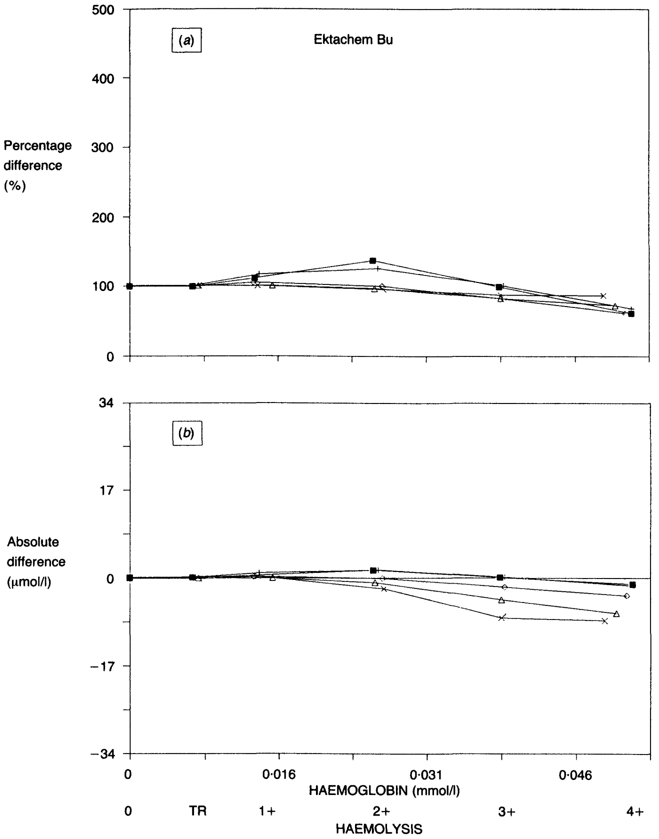

*Figure 7. Effect of increasing amounts of haemolysate on Ektachem Bu results at multiple Bu concentrations. The control (i.e., without added haemolysate) Bu concentrations (µmol/l) were as follows: 3 (■), 5 (+), 10 (♦), 24 (▲), 62 (×). Other details are described in the legend of figure 1.*

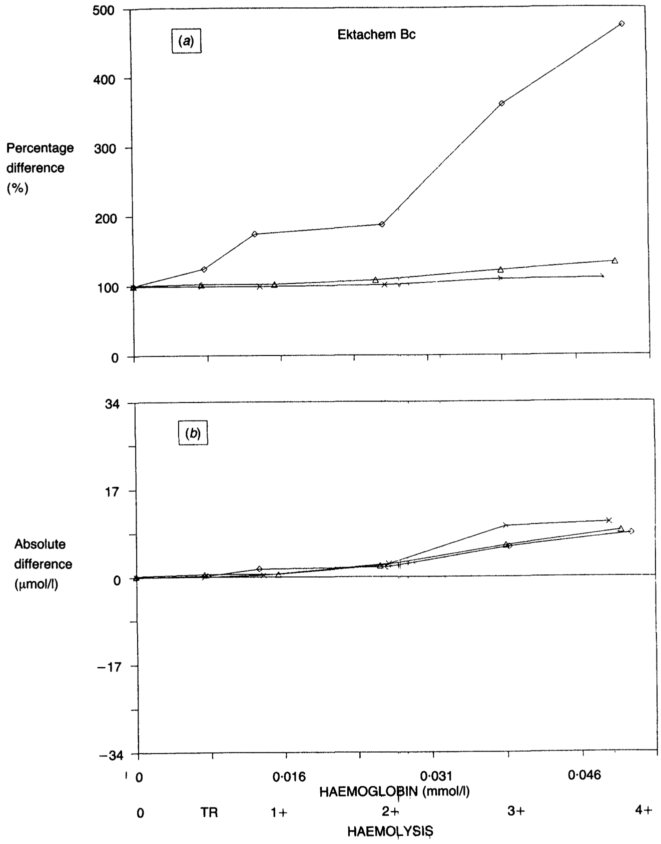

*Figure 8. Effect of increasing amounts of haemolysate on Ektachem Bc results at multiple Bc concentrations. The control (i.e., without added haemolysate) Bc concentrations (µmol/l) were as follows: 3 (♦), 27 (▲), 101 (×). Other details are described in the legend of figure 1.*

DBil assays, it was stated only that haemolysis would interfere with the methods. More information was provided for the Ektachem methods: for the TBil assay, data were presented showing the biases at two concentrations of haemoglobin and one concentration of bilirubin. For the Bu assay, similar data were presented for one concentration of haemoglobin and two concentrations of bilirubin. For the Bc assay, the data were for one concentration of both haemoglobin and bilirubin. Even these data, however, give no indication that the interference (1) can be positive or negative, depending on the relative haemoglobin and bilirubin concentrations; or (2) is neither constant nor proportional with increasing bilirubin concentration.

Glick *et al.* have suggested that interference data be reported in a standardized graphical format as an 'interferograph', where the percentage difference is plotted versus increasing interferant concentration [3]. The authors concur with this but strongly advocate that the data *also* be presented as a plot of the absolute difference versus increasing interferant concentration. Figure 6 demonstrates that this latter format (figure 6[*b*]) can provide a more accurate representation of interference than the former format (figure 6[*a*]). Thus, graphical presentation of interference data as both a percentage-difference interferograph and an absolute-difference interferograph, as done here, would seem to be the best approach.

The fact that all of the automated bilirubin methods tested, except one, exhibited both positive and negative interference, that was neither constant nor proportional, underscores the necessity of doing interference testing at multiple analyte concentrations. Glick and Ryder have presented such data as a three-dimensional interferograph (percentage-difference) [8]. The authors, however, incorporated the additional data into two-dimensional interferographs (percentage- and absolute-difference). Kroll *et al.* have also recognized the importance of using multiple analyte concentrations, and they have described a mathematical model for separately characterizing analyte-dependent and analyte-independent interference [9].

The clinical significance of the results reported here would impinge primarily upon the precise assessment of a patient's course of disease and/or treatment. One potential clinical situation in which the impact could be greater relates to a neonatal jaundice. Assume, for example, that neonatal bilirubin is assayed by a method that exhibits a pattern of haemolysis interference similar to that shown in figure 3, but that the interference is *thought* to be only

*Summary of the type of interference (positive or negative) caused by added haemolysate with different automated bilirubin methods.*

| Method | Low bilirubin Low haemolysate | Low bilirubin High haemolysate | High bilirubin Low haemolysate | High bilirubin High haemolysate |
|---|---|---|---|---|
| SMAC TBil | – | – | + | – |
| Paramax TBil | + | + | – | – |
| Paramax DBil | + | + | – | – |
| Ektachem TBil | + | + | – | – |
| Ektachem DBil | + | + | – | + |
| Ektachem Bu | + | – | – | – |
| Ektachem Bc | + | + | + | + |

positive on the basis of limited interference testing at one bilirubin concentration in the normal range. For a significantly haemolysed neonatal specimen, a bilirubin test result near the level that would indicate therapeutic intervention might be considered falsely elevated, when in fact it would be falsely reduced to a significant degree. This could delay the initiation of treatment; whereas knowledge of the types of results reported here could prevent such delay.

The patterns of haemolysate interference for the automated bilirubin methods examined in this study were complex and depended upon the method, the bilirubin concentration, and the haemoglobin concentration. This suggests that, in general, the most accurate and useful characterization of interference with any automated method is obtained when (1) both the percentage difference and the absolute difference are plotted versus increasing interferant concentration, and (2) the interference is determined at multiple analyte concentrations.

### References

1. Shull, B. C., Lees, H. and Li, P. K., *Clinical Chemistry*, **26** (1980), 22.

2. Shull, B. C., Lees, H. and Li, P. K., *Clinical Chemistry*, **26** (1980), 26.

3. Glick, M. R., Ryder, K. W. and Jackson, S. A., *Clinical Chemistry*, **32** (1986), 470.

4. Frank, J. J., Bermes, E. W., Bickel, M. J. and Watkins, B. F., *Clinical Chemistry*, **24** (1978), 1966.

5. Nelson, D. A. and Morris, M. W. in *Clinical Diagnosis and Management by Laboratory Methods*, 17th edn., Ed. Henry, J. B. (Saunders, Philadelphia, 1984), p. 581.

6. Blakney, G. B. and Dinwoodie, A. J., *Clinical Biochemistry*, **8** (1975), 96.

7. Kahn, S. E., Watkins, B. F. and Bermes, E. W., *Annals of Clinical and Laboratory Science*, **11** (1981), 126.

8. Glick, M. R. and Ryder, K. W., *Clinical Chemistry*, **32** (1986), 1186.

9. Kroll, M. H., Ruddel, M., Blank, D. W. and Elin, R. J., *Clinical Chemistry*, **33** (1987), 1121.

